# Perceived Cognitive Impairment in Breast Cancer Survivors and Its Relationships with Psychological Factors

**DOI:** 10.3390/cancers12103000

**Published:** 2020-10-16

**Authors:** Clémence Boscher, Florence Joly, Bénédicte Clarisse, Xavier Humbert, Jean-Michel Grellard, Giulia Binarelli, Laure Tron, Idlir Licaj, Marie Lange

**Affiliations:** 1 Clinical Research Department, Centre François Baclesse, 14076 Caen, France; f.joly@baclesse.unicancer.fr (F.J.); clarb@baclesse.unicancer.fr (B.C.); JM.GRELLARD@baclesse.unicancer.fr (J.-M.G.); g.binarelli@baclesse.unicancer.fr (G.B.); idlir.licaj@uit.no (I.L.); m.lange@baclesse.unicancer.fr (M.L.); 2 INSERM U1086, ANTICIPE, Normandie Université, UNICAEN, 14000 Caen, France; laure.tron@inserm.fr; 3 Cancer and cognition Platform, Ligue Nationale Contre le Cancer, 14000 Caen, France; 4 Department of Medical Oncology, University Hospital of Caen, 14000 Caen, France; 5 Department of General Medicine, Medical School, 14000 Caen, France; xavier.humbert@unicaen.fr; 6 Department of Pharmacology, University Hospital Caen, 14000 Caen, France; 7 EA4650, Normandie Université, UNICAEN, 14000 Caen, France; 8 Department of Community Medicine, Faculty of Health Sciences, The UiT Arctic University of Norway, 9010 Tromsø, Norway

**Keywords:** cognitive complaints, breast cancer, post-traumatic stress

## Abstract

**Simple Summary:**

Cognitive complaints are common adverse effects for breast cancer survivors, with potential negative impacts on quality of life or return to work. Identifying subjects at risk could allow to reduce cognitive disorders or to set up appropriate care. In this study we explored current cognitive complaints reported by breast cancer survivors, using the Functional Assessment of Cancer Therapy-Cognition (FACT-Cog) questionnaire and examined the relationships between current cognitive complaints and current psychological symptoms (especially post-traumatic stress symptoms). This large survey showed that about half of breast cancer survivors reported cognitive complaints after cancer treatments. These complaints were mainly associated with chemotherapy, age, self-reported sleep difficulties, the frequency of psychotropic treatments and psychological factors including post-traumatic stress symptoms or. Some modifiable risk factors should be detected early to reduce persistent cognitive complaints after cancer, including sleep difficulties and post-traumatic stress symptoms.

**Abstract:**

Cognitive complaints are common adverse effects in cancer patients. Identifying subjects at risk could make it possible to limit their impact. We aimed to explore the relationship between current cognitive complaints and demographic and psychological factors in a group of breast cancer survivors. Through an online survey, cancer survivors reported current cognitive complaints using the FACT-Cog questionnaire (Perceived Cognitive Impairment) and answered questions about their demographics, lifestyle and cancer-related characteristics. Anxiety, depression, fatigue and post-traumatic stress symptoms were also assessed. We used multivariable logistic regression models to explore the relationships between current cognitive complaints and social and psychological factors. Among the 1393 breast cancer survivors, 47.2% (*n* = 657) reported current cognitive complaints. Chemotherapy (OR = 2.26, 95%CI = 1.67–3.05), age (OR_21-44 vs. >65_ = 0.14, 95%CI = 0.07–0.27), sleep difficulties (OR_never vs. often_ = 2.41, 95%CI = 1.47–3.95), frequency of psychotropic treatments (OR_never vs. >1/week_ = 1.70, 95%CI = 1.23–2.36), post-traumatic stress symptoms (OR = 2.05, 95%CI = 1.57–2.69) and employment status (OR_full-time or part-time vs. sick leave_ = 1.64, 95%CI = 1.08–2.49) were strongly associated with current cognitive complaints. In this large study, about half of breast cancer survivors reported cognitive complaints, particularly after chemotherapy. Some risk factors should be detected early to reduce persistent cognitive complaints after cancer: mainly sleep difficulties, post-traumatic stress symptoms and psychotropic medications.

## 1. Introduction

With the recent therapeutic advances in oncology, survival rates are increasing. The quality of life, in particular the “post-cancer” quality of life, is becoming one of the priorities. Cognitive complaints are an adverse effect frequently reported by cancer patients, especially after chemotherapy [1]. Fifteen to fifty percent of breast cancer survivors report cognitive dysfunctions [2]. These disorders can be reported during but also long after cancer diagnosis and treatments, with a negative impact on overall quality of life and ability to return to work [3,4]. Cancer survivors report disorders in several cognitive domains, including difficulties with short-term memory, focusing when performing daily tasks, concentration, word-finding and forgetfulness [5,6].

The mechanisms that contribute to cognitive dysfunction are not yet well-known, but most studies report that they are multifactorial [7]. Cancer treatments are multimodal, and may have a direct effect on cognition, but also interact with various risk factors [8]. Psychological factors can also be associated with cognitive complaints [9]. Anxiety, depression or fatigue are now well-known modifiable risk factors [1,10]. However, the association with post-traumatic stress symptoms has been less studied. To our knowledge, few studies have described this relationship [5], although some results suggest that cognitive dysfunction could be mediated by cancer-related post-traumatic stress [11]. Chapman et al. described an emotional vulnerability with negative effects on perceived cognitive function [12]. Early detection of these psychological factors, which could be modifiable, may allow interventions to reduce them and improve cognitive complaints.

In our previous study using a web-based survey, 75% (*n* = 1214) of the 1610 participants (rate of breast cancer = 87%) reported experiencing cognitive complaints related to cancer treatments (past or current at the time of the study) based on a single question [13]. Chemotherapy, age, self-reported sleep difficulties and pre-existing knowledge about chemotherapy-associated cognitive problems were strongly associated with cancer-related experience of cognitive complaints.

In the present study, based on the same survey, we explored current and significant cognitive complaints reported only by the subgroup of breast cancer survivors and using the Functional Assessment of Cancer Therapy-cognitive function (FACT-Cog) instead of a simple closed question [14]. We also examined the relationships between current cognitive complaints and current psychological symptoms (especially post-traumatic stress symptoms). We hypothesized that chemotherapy, age, self-reported sleep difficulties, pre-existing knowledge about chemotherapy-associated cognitive problems and current psychological factors, including post-traumatic stress symptoms, would be associated to current cognitive complaints.

## 2. Results

### 2.1. Participant Characteristics

The sample included 1393 breast cancer survivors. The median age was 52 years (25–84 years). Two-thirds of breast cancer survivors had a high education level, and a large majority of participants had a localized cancer (Table 1).

Concerning cancer-related treatments, 1065 (76.5%) participants received chemotherapy, 1039 (74.6%) were treated by endocrine therapy and 133 (9.6%) by targeted therapy. Median post-cancer curative treatment time was 2.58 years (0.08–33 years).

Concerning psychological factors, 472 (33.9%) breast cancer survivors reported significant anxiety symptoms and 131 (9.4%) reported significant symptoms of depression according to the Hospital Anxiety and Depression Scale (HADS) scores. Significant fatigue (FACIT-F, Functional Assessment of Chronic Illness Therapy-Fatigue) was reported by 115 participants (8.3%) and post-traumatic stress symptoms, as evaluated by the Impact of Event Scale-Revised (IES-R), were reported by 386 participants (27.7%).

### 2.2. Univariable Analyses

#### 2.2.1. Questionnaires and Associated Factors

Descriptive statistics for the four subscales of FACT-Cog, the HADS (depression and anxiety), the ES-R, and the FACIT-F scores are presented in Table 2.

In a first univariable analysis, we observed that the four FACT-Cog scores were strongly associated with age, employment status, self-reported sleep difficulties, frequency of psychotropic treatments, self-reported cancer-related cognitive complaints, chemotherapy, anxiety, depression, fatigue and post-traumatic stress symptoms (*p* < 0.001, Table S1). There was no relationship with body mass index (BMI), education level, marital status, physical activity, history of neurological disease, pre-existing knowledge about chemotherapy-associated cognitive problems, metastatic status, targeted therapy or endocrine therapy (Table S1). PCI (Perceived Cognitive Impairment) was the only FACT-Cog sub-scale to be associated with post-cancer curative treatment time (*p* < 0.001, Table S1).

#### 2.2.2. Current Cognitive Complaints

**Figure 1:**
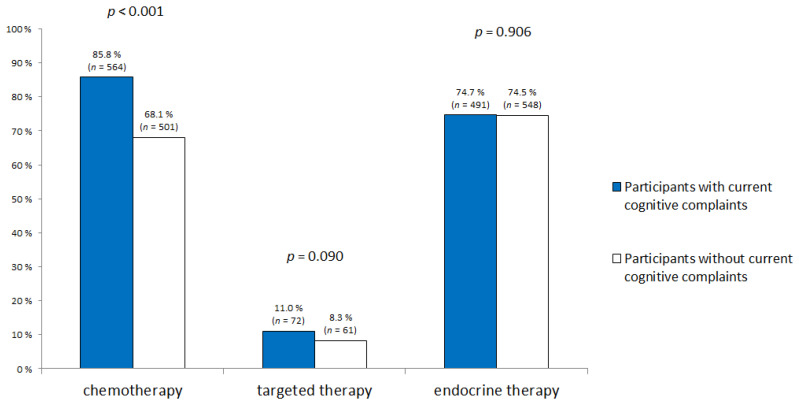
Cancer-related treatments according to current cognitive complaints.

**Figure 2:**
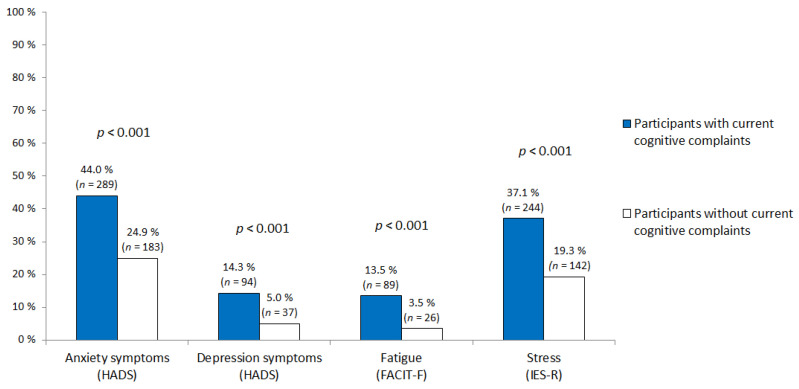
Significant psychological symptoms (anxiety, depression, fatigue and post-traumatic stress symptoms) according to current cognitive complaints. (Abbreviations = HADS: Hospital Anxiety and Depression Scale; IES-R: Impact of Event Scale-Revised; FACIT-F: Functional Assessment of Chronic Illness Therapy-Fatigue.).

Forty-seven percent of the participants (*n* = 657) had current cognitive complaints at the time of the survey. Compared with participants who had no current cancer-related cognitive complaints, those who had current complaints were younger (*p* < 0.001, Table 1) and more likely to be on sick leave (*p* < 0.001, Table 1). Current cognitive complaints were also associated with post-cancer curative treatment time (*p* < 0.001), frequency of psychotropic treatments (*p* < 0.001), self-reported sleep difficulties (*p* < 0.001) and pre-existing knowledge about chemotherapy-associated cognitive problems (*p* < 0.001). Participants with current cognitive complaints received chemotherapy more often (*p* < 0.001, Figure 1). They also reported more anxiety, depression, fatigue and post-traumatic stress symptoms than participants without current cognitive complaints (*p* < 0,001, Figure 2). However, education level, marital status, metastatic status, BMI, frequency of physical activity and history of neurological disease were not associated with current cancer-related cognitive complaints.

### 2.3. Multivariable Analyses

Multivariable analyses were based on three models: (1) chemotherapy, (2) chemotherapy + IES-R, (3) endocrine therapy. Regardless of the model used, the relationships with different demographic, lifestyle or cancer-related characteristics were similar (Table 3).

In the first model, participants who received chemotherapy had higher odds of cognitive complaints compared with those who did not (OR = 2.26, 95% CI = 1.67–3.05). The odds of presenting current cognitive complaints were decreased for older survivors (OR_21–44 vs. >65_ = 0.14, 95% CI = 0.07–0.27). Employment status (OR_full-time or part-time vs. sick leave_ = 1.64, 95% CI = 1.08–2.49), self-reported sleep difficulties (OR_never vs. often_ = 2.41, 95% CI = 1.47–3.95) and frequency of psychotropic treatments (OR_never vs. >1/week_ = 1.70, 95% CI = 1.23–2.36) were also associated with current cognitive complaints. In contrast, no association was found with post-cancer curative treatment time, metastatic status, education level, physical activity or pre-existing knowledge about chemotherapy-associated cognitive problems.

In the second model, post-traumatic stress symptoms were strongly associated with current cognitive complaints (OR = 2.05, 95% CI = 1.57–2.69).

A sensitivity multivariable analysis using the same covariates, but based on endocrine therapy rather that chemotherapy, showed similar results (Table 3). No association was found with endocrine therapy either for current or former users compared with never users (OR_never vs. current_ = 1.11, 95% CI = 0.84–1.47). However, pre-existing knowledge about chemotherapy-associated cognitive problems was associated with current cognitive complaints in this model (OR = 1.35, 95% CI = 1.07–1.71).

## 3. Discussion

Using a validated self-report questionnaire and a large sample, this survey showed that about half of breast cancer survivors had current cognitive complaints at the time of the study, after cancer treatments. These complaints were mainly associated with age, chemotherapy, self-reported sleep difficulties and psychological factors, including post-traumatic stress symptoms or frequency of psychotropic treatments.

Our results are in line with previous studies, which reported that 16% to 60% of cancer survivors had cognitive complaints [15,16]. Chemotherapy was strongly associated with cognitive complaints, even after a few years of treatment. The odds of developing cognitive complaints is about twice as high (95% confidence interval = 1.67–3.05) in patients who have been treated with chemotherapy—a phenomenon known as “chemobrain”. Our results are in line with previous studies [2,16,17,18,19,20]. In a recent prospective longitudinal study, cancer survivors treated with chemotherapy reported more cognitive complaints than matched healthy controls [9]. Chemotherapy induced subjective cognitive impairment was not only more frequent, but also more severe than in patients not treated with chemotherapy [1]. In the TAILORx study, Wagner et al. reported that cognitive subjective decline secondary to chemotherapy seemed to be early and acute, but did not worsen over time [21]. Many complex mechanisms are implicated, including direct neurotoxic effects induced by chemotherapy (which can cross the blood-brain barrier), and indirect impacts caused by pro-inflammatory cytokine levels as well as some genetic polymorphisms [22].

Very few studies have assessed the impact of post-traumatic stress symptoms on cognition in cancer patients. Hermelink et al. showed that before any treatment, breast cancer patients may have cognitive deficiency caused by post-traumatic stress related to cancer [23]. A second study of these authors found that cognitive dysfunction in breast cancer survivors was mediated by cancer-related post-traumatic stress (*n* = 226) [11]. Our results are in line with these previous studies. Using a validated self-report questionnaire and a large sample (*n* = 1393), we found that post-traumatic stress symptoms were strongly associated with cognitive complaints among breast cancer survivors. The odds that participants reported cognitive disorders were between 1.57 and 2.69 higher when they also reported significant post-traumatic stress symptoms. Stressful exposures like disease may block the neurogenesis of the hippocampus, which is strictly related to memory. This is perhaps due to altered expression of the GABA-A receptors involved in the development of pathological anxiety and various mood disorders [24].

Cognitive difficulties were greater in participants using psychotropic treatments. Furthermore, self-reported sleep difficulties were strongly associated with cognitive complaints. We found that the odds of reporting cognitive complaints were between 1.47 and 3.95 higher when sleep difficulties were reported. This finding is in line with previous studies [10]. Sleep disturbance is known to be a major factor of cognitive decline [25], and may be improved by medicinal or para-medical interventions. Indeed, most current pharmaceutical approaches tend to only improve symptoms temporarily, without treating the underlying etiologies. Exercise and behavioral interventions are increasingly being recognized as effective in managing chronic symptoms, and possibly in treating an underlying cause [26].

After adjustments, pre-existing knowledge about chemotherapy-associated cognitive dysfunction was associated with cognitive complaints in the model with endocrine therapy. Clear, reliable information and reassurance could therefore make it possible to reduce the risk for patients who are worried about this, since they had made inquiries beforehand. However, patients should receive the appropriate type of information so that they are not unduly worried. In fact, in a previous study, simple information about the relationship between cognitive problems and chemotherapy may have impacted patients’ cognitive problems negatively and reduced their cognitive performance—particularly in patients who had previous experience of chemotherapy [27].

In some studies, younger participants had more cognitive complaints than older patients, perhaps due to the fact that older patients did not associate their cognitive complaints with cancer treatment, but rather with normal aging [5,20,28]. Moreover, younger patients are often well informed, thanks to new information media such as the Internet. Life cycle and cognitive challenges (due to family or work) could also be associated with younger patients, thus younger patients may note more issues with cognitive changes.

The use of endocrine therapy was also strongly associated with current cognitive complaints in the univariable analysis. However, a significant relationship was not found after multivariable correction. The role of endocrine therapy is still debated [21]. Some studies have shown endocrine therapy to have detrimental effects on some neuropsychological tests [29,30,31]. Indeed, the role of estrogens in neuro-protection and cognitive functioning is now well known [32]. However, the effects differ depending on the drug class, which was not specified in our survey. For example, tamoxifen was associated with worse neuropsychological impairment, whereas exemestane was not [33].

In our study, participants with cognitive complaints were more often on sick leave, a side-effect that has an impact on work resumption [34]. Survivors may be on sick leave due to cognitive impairment, but failure to return to work may also increase cognitive complaints. In a recent study, Von Ah et al. showed that PCI was associated with poorer work ability [35]. Work means a return to the usual activities of life, so early rehabilitation can help to limit the cognitive impairment of cancer survivors.

In contrast with some publications, physical activity was not associated with cognitive complaints in the present study [5,13]. We hypothesized that participants with current cognitive complaints were more often on sick leave, and so could practice fewer activities due to current cancer treatments.

Our study has some limitations. First, it was a web-based survey and the voluntary nature of the participation process may have created a selection bias. Indeed, patients with cognitive difficulties were more likely to answer this survey, which may have led to an overestimation of the number of participants with complaints. Furthermore, the participants may not be representative of the usual population of cancer survivors. In fact, breast cancer survivors who participate in social networks may have a particular (especially socio-demographic) profile. Furthermore, we did not have a control group. Finally, we explored the cognitive complaints of participants at a specific point in time, so the time between the end of treatment and the survey was not the same for all participants.

## 4. Materials and Methods

### 4.1. Procedure

The procedure of this web-based survey is described elsewhere [13]. Participants were enrolled through the national cancer patient network, called “Seintinelles”, from October, 2016, to October, 2017. The previous article probed cognitive side-effects with the question: “Have you (had) memory, concentration, finding words or other cognitive difficulties during or after cancer treatments?” [13]. These results included cognitive complaints experienced (current at the time of the study or past experience). The present study focused on the analysis of current and significant cognitive complaints during survey participation using the validated self-report questionnaire FACT-Cog based on the last seven days [14].

### 4.2. Participants

Eligible participants were French adult cancer survivors who had finished their curative treatment (participation was possible if endocrine therapy was ongoing) [13]. The exclusion criteria were history of progressive neurological or psychiatric disease and drug abuse. All participants gave their consent on the survey website.

The study was approved by the French Advisory Committee on treatment of research information (file N°16.027, approved 13 January 2016) and the Data Protection Agency (authorization DR-2016-2016-340, 21 July 2016). For this single-center observational study, according to French legislation in 2016, no ethics committee approval was needed. Only the approval for the processing of personal data was required by the French Advisory Committee on treatment of research information and the Data Protection Agency.

As they constituted the large majority of participants and in order to homogenize the study population, we only included patients treated for breast cancer in the present paper (*n* = 1393, 87%).

### 4.3. Assessment

The variables which were collected included demographic variables (age, education level, marital and employment status, BMI, history of neurological disease), lifestyle characteristics (sleep difficulties, physical activity, frequency of psychotropic treatments) and cancer-related characteristics (metastatic status, cancer treatments, time since the end of curative treatment, excluding endocrine therapy). Pre-existing knowledge about chemotherapy-associated cognitive problems was also evaluated. Participants performed four self-report questionnaires.

The FACT-Cog is a self-report questionnaire with 37 items. It assesses cognitive complaints over the last seven days [14,36]. The FACT-Cog is the only self-report measure specific to cancer patients’ cognitive function that has been validated in French. The patient has to indicate how often the situation occurred during the last seven days. The higher the score, the lower the cognitive complaints. The FACT-Cog includes four subscales: Perceived Cognitive Impairment (PCI), Comments from Others (Oth), Perceived Cognitive Abilities (PCA) and impact on Quality of Life (QoL).

The IES-R is a 22-item self-report measure of post-traumatic stress symptoms. Symptoms related to cancer were assessed during the last seven days [37]. Significant post-traumatic stress symptoms were defined as a score equal to or greater than 33 [38].

The FACIT-F is a 13-item measure that asks respondents to rate symptoms of fatigue during the last seven days [39]. Higher scores reflect lower fatigue. Significant fatigue was defined by a score lower than 37 [40].

The HADS is a 14-item self-report scale [41] validated in French for cancer patients [42]. It includes seven items on anxiety assessed during the last seven days (HADS-anxiety) and seven items on depression during the last seven days (HADS-depression). Significant anxiety symptoms were defined by a score equal to or greater than 11 and significant depression symptoms were defined by a score equal to or greater than 11.

### 4.4. Statistical Analysis

For the present study, the PCI score was chosen to define current cognitive complaints because it was the most used of the four FACT-Cog subscales as a primary outcome of cognitive complaints in previous studies [21] and can be considered as a separate score from the other subscales [43].

More precisely, current and significant cognitive complaints were defined by a response “Yes” to the question “Have you (had) memory, concentration, finding words or other cognitive difficulties during or after cancer treatments?”, and by PCI significant cognitive complaints at the time of the survey, according to normative data based on age (operationally defined as ratings ≤ 10th percentile of the PCI scores) [44].

Descriptive statistics were generated for all questionnaires. Participants, treatments and other socio-demographic characteristics were compared between survivors who had current cognitive complaints and those who did not. We used the standard Chi-squared test (Fisher’s exact test when appropriate), analysis of variance (ANOVA), or the Wilcoxon or Kruskal–Wallis tests.

Three multivariable logistic regression models were run to compute the odds ratios (ORs) of cognitive complaints and their 95% confidence intervals (CIs) [45]. In the first model, we adjusted for all covariates included in the multivariable model of the previous manuscript [13] in order to compare the ORs precisely between the two studies. The list of covariates included: chemotherapy (yes; no), post-cancer curative treatment time (≤1 years; 1–3 years; ≥3 years), metastatic status (yes; no), age (21–44; 45–64; ≥65 years), employment status (employed (full-time or part-time); sick leave; student or retired; unemployed; other), education level (low; middle; high), self-reported sleep difficulties (never; sometimes; often), frequency of psychotropic treatments (never; <1/month; ≥1/month and <1/week; ≥1/week), physical activity (none or < once a week; once a week; twice a week; ≥3 times a week), and pre-existing knowledge about chemotherapy-associated cognitive problems (yes; no).

The second multivariable model also included post-traumatic stress symptoms through the IES-R (yes; no). We focused on post-traumatic stress because few studies have been made of its relationship with cognitive complaints [23]. We therefore chose not to include anxiety, depression or fatigue in our multivariable models since these variables are highly associated with each other, and with post-traumatic stress symptoms.

The third model included endocrine therapy (never; former; current) rather than chemotherapy.

To minimize the risk of false-positive results, only associations with a *p*-value ≤ 0.01 were considered as statistically significant.

The Bayesian information criterion was used to compare the model fit. All statistical analyses were performed using STATA version 15 (Stata Corp, College Station, TX, USA).

## 5. Conclusions

Overall, one in two patients reported cognitive complaints. These were strongly associated with chemotherapy as well as with multiple modifiable risk factors such as post-traumatic stress, anxiety, depression and sleep difficulties. Identifying subjects at risk could therefore make it possible to manage these factors in order to reduce cognitive complaints and to set up appropriate care, thus limiting the cognitive difficulties of these patients and their impact on quality of life.

## Figures and Tables

**Table 1:** Demographic, clinical and lifestyle characteristics of participants according to current cognitive complaints.

*N* (%)	All Sample	Participants with Current Cognitive Complaints (*n* = 657)	Participants without Current Cognitive Complaints (*n* = 736)	*p*
Age in years				
21–44	322 (23.1%)	226 (34.4%)	96 (13.0%)	**<0.001**
45–64	940 (67.5%)	413 (62.9%)	527 (71.6%)	
≥65	131 (9.4%)	18 (2.7%)	113 (15.4%)	
Education level				
Low	44 (3.2%)	17 (2.6%)	27 (3.7%)	0.284
Middle	373 (26.8%)	168 (25.6%)	205 (27.8%)	
High	976 (70.0%)	472 (71.8%)	504 (68.5%)	
Employment				
Full-time or part-time	787 (56.5%)	384 (58.5%)	403 (54.8%)	**<0.001**
Sick leave	148 (10.6%)	100 (15.2%)	48 (6.5%)	
Student, retired	249 (17.9%)	58 (8.8%)	191 (25.9%)	
Unemployed	64 (4.6%)	39 (5.9%)	25 (3.4%)	
Other	145 (10.4%)	76 (11.6%)	69 (9.4%)	
Married/partnered				
No	364 (26.1%)	159 (24.2%)	205 (27.9%)	0.121
Yes	1029 (73.9%)	498 (75.8%)	531 (72.1%)	
Cancer without metastasis				
Yes	1236 (88.7%)	575 (87.5%)	661 (89.8%)	0.177
No	157 (11.3%)	82 (12.5%)	75 (10.2%)	
Post-cancer curative treatment time				
≤1 year	373 (26.8%)	210 (32.0%)	163 (22.1%)	**<0.001**
1–3 years	336 (24.1%)	167 (25.4%)	169 (23.0%)	
≥3 years	684 (49.1%)	280 (42.6%)	404 (54.9%)	
BMI				
Insufficient	51 (3.7%)	30 (4.6%)	21 (2.9%)	0.037
Normal	802 (57.6%)	377 (57.4%)	425 (57.7%)	
Overweight	377 (27.0%)	162 (24.6%)	215 (29.2%)	
Obesity	163 (11.7%)	88 (13.4%)	75 (10.2%)	
Frequency of psychotropic treatments				
Never	914 (65.6%)	389 (59.2%)	525 (71.3%)	**<0.001**
<1/month	169 (12.1%)	91 (13.8%)	78 (10.6%)	
>1/month and <1/week	77 (5.5%)	38 (5.8%)	39 (5.3%)	
>1/week	233 (16.8%)	139 (21.2%)	94 (12.8%)	
Self-reported sleep difficulties				
Never	95 (6.8%)	34 (5.2%)	61 (8.3%)	**<0.001**
Sometimes	481 (34.5%)	197 (30.0%)	284 (38.6%)	
Often	817 (58.7%)	426 (64.8%)	391 (53.1%)	
Physical activity				
None or < once a week	446 (32.0%)	203 (30.9%)	243 (33.0%)	0.431
Once a week	255 (18.3%)	116 (17.7%)	139 (18.9%)	
Twice a week	362 (26.0%)	184 (28.0%)	178 (24.2%)	
≥3 times a week	330 (23.7%)	154 (23.4%)	176 (23.9%)	
History of neurological disease				
No	1316 (94.5%)	625 (95.1%)	691 (93.9%)	0.311
Yes	77 (5.5%)	32 (4.9%)	45 (6.1%)	
Pre-existing knowledge ^1^				
No	842 (60.5%)	359 (54.6%)	483 (65.6%)	**<0.001**
Yes	551 (39.5%)	298 (45.4%)	253 (34.4%)	

^1^ Pre-existing knowledge about chemotherapy-associated cognitive problems. In bold in the table: significant results.

**Table 2:** Descriptive data for scores of questionnaires.

Questionnaires		Median	Mean	SD
Cognitive complaints (FACT-Cog)	PCI (0–72)	50	48.1	15.6
	PCA (0–28)	15	15.6	5.6
	Oth (0–16)	16	14.4	2.6
	QoL (0–16)	8	8.8	4.7
HADS	Depression (0–21)	5	5.4	3.5
	Anxiety (0–21)	8	8.9	4.0
Post-traumatic stress symptoms (IES-R) (0–88)		21	23.2	16.0
Fatigue (FACIT-F) (0–52)		19	19.5	11.8

(Abbreviations: FACT-Cog: Functional assessment of cancer therapy-cognitive function; PCI: Perceived Cognitive Impairment; PCA: Perceived Cognitive Abilities; Oth: Comments from Others; QoL: impact on Quality of Life; HADS: Hospital Anxiety and Depression Scale; IES-R: The Impact of Event Scale-Revised; FACIT-F: The Functional Assessment of Chronic Illness Therapy-Fatigue).

**Table 3:** Multivariable odds ratios (ORs) and 95% confidence intervals (CIs) of current cancer cognitive complaints and clinical, demographic and lifestyle characteristics based on 3 models: (1) chemotherapy, (2) chemotherapy + IES-R (Impact of Event Scale-Revised), (3) endocrine therapy.

Multivariable	1 = Chemotherapy		2 = IES-R		3 = Endocrine Therapy	
Mean (SD)	OR	95% CI	OR	95% CI	OR	95% CI
Chemotherapy						
No	1.00	Reference	1.00	Reference		
Yes	**2.26**	**1.67–3.05**	**2.28**	**1.68–3.08**		
Post-cancer curative treatment time in years						
≤1	1.00	Reference	1.00	Reference	1.00	Reference
1–3	0.98	0.70–1.37	1.00	0.71–1.41	1.05	0.75–1.46
≥3	0.79	0.59–1.08	0.83	0.61–1.12	0.89	0.66–1.22
Cancer without metastasis						
Yes	1.00	Reference	1.00	Reference	1.00	Reference
No	1.04	0.72–1.50	1.04	0.72–1.51	1.23	0.86–1.77
Age in years						
21–44	1.00	Reference	1.00	Reference	1.00	Reference
45–64	**0.37**	**0.28–0.51**	**0.37**	**0.27–0.50**	**0.33**	**0.24–0.44**
≥65	**0.14**	**0.07–0.27**	**0.15**	**0.07–0.30**	**0.11**	**0.05–0.21**
Employment						
Full-time or part-time	1.00	Reference	1.00	Reference	1.00	Reference
Sick leave	**1.64**	**1.08–2.49**	**1.57**	**1.03–2.52**	**1.67**	**1.11–2.52**
Student/retired	0.69	0.45–1.04	0.67	0.44–1.03	0.70	0.46–1.05
Unemployed	1.62	0.93–2.84	1.43	0.81–2.52	1.69	0.97–2.94
Other	1.24	0.84–1.82	1.18	0.80–1.75	1.25	0.86–1.83
Education level						
Low	1.00	Reference	1.00	Reference	1.00	Reference
Middle	1.02	0.50–2.07	1.02	0.50–2.10	1.05	0.52–2.13
High	1.11	0.55–2.23	1.13	0.56–2.28	1.13	0.57–2.25
Self-reported sleep difficulties						
Never	1.00	Reference	1.00	Reference	1.00	Reference
Sometimes	1.51	0.91–2.49	1.41	0.85–2.33	1.51	0.92–2.49
Often	**2.41**	**1.47–3.95**	**2.08**	**1.26–3.42**	**2.34**	**1.43–3.84**
Frequency of psychotropic treatments						
Never	1.00	Reference	1.00	Reference	1.00	Reference
<1/month	**1.56**	**1.08–2.26**	**1.51**	**1.04–2.19**	**1.52**	**1.06–2.18**
>1/month and <1/week	1.23	0.73–2.07	1.04	0.61–1.77	1.16	0.70–1.93
>1/week	**1.70**	**1.23–2.36**	**1.60**	**1.15–2.23**	**1.69**	**1.23–2.34**
Physical activity						
None or <once a week	1.00	Reference	1.00	Reference	1.00	Reference
Once a week	0.92	0.66–1.30	0.92	0.65–1.29	0.92	0.66–1.30
Twice a week	1.30	0.95–1.76	1.28	0.94–1.74	1.31	0.96–1.77
≥3 times a week	1.08	0.78–1.48	1.11	0.80–1.53	1.10	0.80–1.51
Pre-existing knowledge ^1^						
Yes	1.00	Reference	1.00	Reference	1.00	Reference
No	1.23	0.96–1.56	1.25	0.98–1.59	**1.35**	**1.07–1.71**
Stress symptoms (IES-R)						
No			1.00	Reference		
Yes			**2.05**	**1.57–2.69**		
Endocrine therapy						
Never					1	Reference
Former					0.96	0.67–1.39
Current					1.11	0.84–1.47

^1^ Pre-existing knowledge about chemotherapy-associated cognitive problems. In bold in the table: significant results.

## Supplementary Materials

The following are available online at https://www.mdpi.com/2072-6694/12/10/3000/s1. Table S1: Relationships between FACT-Cog subscales and demographic, clinical, psychological and lifestyle characteristics.
