# Supplementary material for: Perceived Cognitive Impairment in Breast Cancer Survivors and Its Relationships with Psychological Factors
Source: Cancers (Basel). 2020 Oct 16;12(10):3000. doi: 10.3390/cancers12103000 (PMC7602817; doi:10.3390/cancers12103000)
Supplement: Supplementary file 1 [file cancers-12-03000-s001.pdf]

Supplementary Materials:

Perceived Cognitive Impairment in Breast Cancer Survivors and Its Relationships with Psychological Factors

Clémence Boscher, Florence Joly, Bénédicte Clarisse, Xavier Humbert, Jean-Michel Grellard, Giulia Binarelli, Laure Tron, Idlir Licaj and Marie Lange

Table S1. Relationships between FACT-Cog subscales and demographic, clinical, psychological and lifestyle characteristics.

Mean (SD)	PCI	p	PCA	p	Oth	p	QoL	p
Age in years								
21–44	46.3 (16.2)	**<0.001**	14.9 (5.7)	**<0.001**	14.2 (2.6)	**<0.001**	8.5 (4.6)	**<0.001**
45–64	47.8 (15.6)		15.5 (5.6)		14.3 (2.7)		8.6 (4.7)	
≥65	55.1 (12.1)		17.7 (5.2)		15.2 (1.9)		11.5 (4.0)	
BMI								
Insufficient	46.2 (15.7)	0.4902	15.1 (5.7)	0.4364	14.4 (2.3)	0.9404	8.7 (4.8)	0.2189
Normal	48.5 (15.7)		15.7 (5.6)		14.4 (2.6)		9.0 (4.7)	
Overweight	48.1 (15.4)		15.5 (5.4)		14.4 (2.4)		8.9 (4.7)	
Obesity	46.9 (15.9)		15.1 (5.9)		14.3 (2.8)		8.1 (4.8)	
Education level								
Low	50.4 (16.4)	0.3826	15.5 (6.6)	0.065	14.8 (2.5)	0.1537	9.7 (5.0)	0.3893
Middle	47.7 (15.2)		14.9 (5.4)		14.3 (2.7)		8.7 (4.6)	
High	48.2 (15.8)		15.8 (5.6)		14.4 (2.5)		8.8 (4.8)	
Employment								
Full-time or part-time	48.3 (15.6)	**<0.001**	15.8 (5.7)	**<0.001**	14.4 (2.4)	**<0.001**	8.8 (4.6)	**<0.001**
Sick leave	42.1 (15.8)		13.5 (4.8)		13.6 (3.2)		7.3 (4.4)	
Student, retired	53.1 (13.8)		16.9 (5.2)		15.0 (1.9)		10.8 (4.4)	
Unemployment	43.2 (16.8)		14.1 (5.9)		13.2 (3.9)		7.0 (4.7)	
Other	46.7 (15.5)		15.0 (6.1)		14.4 (2.5)		8.0 (4.9)	
Married/partnered								
No	48.0 (16.2)	0.8921	15.6 (5.8)	0.8761	14.4 (2.8)	0.0557	8.7 (4.9)	0.5222
Yes	48.1 (15.4)		15.6 (5.6)		14.4 (2.5)		8.9 (4.7)	
Self-reported sleep difficulties								
Never	54.0 (15.7)	**<0.001**	17.8 (6.6)	**<0.001**	14.8 (2.2)	**<0.001**	10.5 (5.0)	**<0.001**
Sometimes	51.1 (14.6)		16.5 (5.6)		14.7 (2.2)		9.9 (4.5)	
Often	45.7 (15.8)		14.8 (5.4)		14.1 (2.8)		8.0 (4.6)	
Physical activity								
None or <once a week	48.1 (15.8)	0.7645	15.7 (5.8)	0.1888	14.5 (2.3)	0.5247	8.8 (4.7)	0.5876
Once a week	48.8 (14.7)		15.2 (5.5)		14.3 (2.6)		8.7 (4.7)	
Twice a week	47.4 (15.6)		15.2 (5.4)		14.3 (2.7)		8.7 (4.5)	
≥3 times a week	48.4 (16.1)		16.1 (5.7)		14.3 (2.7)		9.2 (5.0)	
Frequency of psychotropic treatments								
Never	50.2 (15.1)	**<0.001**	16.2 (5.6)	**<0.001**	14.6 (2.4)	**0.0017**	9.6 (4.6)	**<0.001**
<1/month	44.9 (14.9)		14.6 (4.9)		14.3 (2.4)		8.0 (4.6)	
>1/month and <1/week	45.9 (15.3)		14.9 (5.4)		14.1 (2.9)		7.9 (4.4)	
>1/week	42.8 (16.7)		13.8 (5.7)		13.8 (3.2)		6.9 (4.6)	
History of neurological disease								
No	48.1 (15.6)	0.8374	15.6 (5.6)	0.989	14.4 (2.6)	0.5945	8.8 (4.7)	0.071
Yes	47.9 (15.6)		15.4 (6.3)		14.5 (2.0)		8.7 (4.8)	
Cancer-related cognitive complaints[1]								
No	60.4 (11.0)	**<0.001**	20.0 (5.3)	**<0.001**	15.5 (1.5)	**<0.001**	13.0 (3.7)	**<0.001**
Yes	44.3 (14.9)		14.2 (5.0)		14.1 (2.7)		7.6 (4.2)	
Pre-existing knowledge[2]								
No	48.9 (15.3)	0.0268	15.7 (5.6)	0.3053	14.4 (2.5)	0.622	9.1 (4.7)	**0.0066**
Yes	46.9 (16.0)		15.3 (5.6)		14.3 (2.7)		8.4 (4.8)	
Postcancer curative treatment time								

≤1 year	45.9 (15.0)	**<0.001**	15.1 (5.3)	0.0867	14.2 (2.6)	0.1215	8.6 (4.4)	0.2856
1–3 years	47.8 (16.3)		15.6 (5.7)		14.4 (2.6)		8.9 (4.7)	
≥3 years	49.5 (15.3)		15.8 (5.8)		14.5 (2.5)		9.0 (4.9)	
Cancer without metastasis								
Yes	48.3 (15.7)	0.0743	15.6 (5.7)	0.1945	14.4 (2.6)	0.5765	8.9 (4.8)	0.2894
No	46.5 (14.7)		14.9 (5.3)		14.4 (2.2)		8.4 (4.4)	
Chemotherapy								
No	51.6 (15.0)	**<0.001**	16.7 (5.6)	**<0.001**	14.7 (2.2)	**0.0042**	9.7 (4.6)	**<0.001**
Yes	47.0 (15.7)		15.2 (5.6)		14.3 (2.7)		8.6 (4.7)	
Targeted therapy								
No	48.1 (15.6)	0.8957	15.6 (5.6)	0.9018	14.4 (2.5)	0.5844	8.8 (4.7)	0.5668
Yes	48.0 (15.9)		15.4 (5.7)		14.0 (3.1)		9.0 (5.2)	
Endocrine therapy								
No	48.7 (16.1)	0.3013	15.6 (6.0)	0.9376	14.4 (2.6)	0.6037	9.2 (4.8)	0.0854
Yes	47.9 (15.5)		15.6 (5.5)		14.4 (2.6)		8.7 (4.7)	
Endocrine therapy								
Never	48.7 (16.1)	0.0404	15.6 (6.0)	0.4425	14.4 (2.6)	0.2545	9.2 (4.8)	0.1535
Former	50.1 (13.9)		15.9 (5.3)		14.6 (2.3)		8.9 (4.8)	
Current	47.2 (15.9)		15.4 (5.5)		14.3 (2.7)		8.6 (4.7)	
HADS anxiety								
Yes	42.1 (15.5)	**<0.001**	13.5 (5.0)	**<0.001**	13.6 (3.1)	**<0.001**	6.8 (4.3)	**<0.001**
No	51.2 (14.8)		16.6 (5.6)		14.8 (2.2)		9.9 (4.6)	
HADS depression								
Yes	35.3 (15.7)	**<0.001**	11.2 (4.7)	**<0.001**	13.1 (3.3)	**<0.001**	5.2 (4.0)	**<0.001**
No	49.4 (15.0)		16.0 (5.5)		14.5 (2.5)		9.2 (4.6)	
Fatigue (FACIT-F)								
Yes	29.8 (15.4)	**< 0.001**	11.2 (4.5)	**< 0.001**	12.2 (3.9)	**<0.001**	3.6 (3.3)	**<0.001**
No	49.8 (14.6)		16.0 (5.6)		14.6 (2.3)		9.3 (4.5)	
Post-traumatic stress symptoms (IES-R)								
Yes	41.1 (15.4)	**<0.001**	13.2 (4.9)	**<0.001**	13.3 (3.2)	**<0.001**	6.4 (4.2)	**<0.001**
No	50.8 (14.9)		16.5 (5.6)		14.8 (2.2)		9.8 (4.6)	

[1] Self-report cancer-related cognitive complaints through the following question, "Have you (had) memory, concentration, findings words or other cognitive difficulties during of after cancer treatments?", [2] Pre-existing knowledge about chemotherapy-associated cognitive problems, (Abbreviations = FACT-Cog: Functional assessment of cancer therapy-cognitive function; PCI: Perceived Cognitive Impairments; PCA: Perceived Cognitive Abilities; Oth: Comments From Others; QoL: impact on Quality of Life; HADS: Hospital Anxiety and Depression Scale; IES-R: The Impact of Event Scale-Revised; FACIT-F: The Functional Assessment of Chronic Illness Therapy-Fatigue.). In bold in the table: significant results.
